# Vehicle Company’s Decision-Making to Process Waste Batteries: A Game Research under the Influence of Different Government Subsidy Strategies

**DOI:** 10.3390/ijerph192113771

**Published:** 2022-10-23

**Authors:** Menglin Zhan, Yan Chen

**Affiliations:** 1 College of Economics and Management, Nanjing Forestry University, Nanjing 210037, China; 2 Academy of Chinese Ecological Progress and Forestry Development Studies, Nanjing Forestry University, Nanjing 210037, China

**Keywords:** waste battery, subsidy strategy, processing decision, game model

## Abstract

With the increase in the number of waste power batteries and the occurrence of related environmental problems, battery recycling is receiving extensive attention. Driven by economic benefits, many companies have begun to deploy the waste battery processing market and government subsidies also play an essential role in battery recycling. Considering the vehicle company outsources processing tasks or invests in research and development (R&D), this paper studies the optimal decision-making problem of the supply chain under government subsidy to the battery manufacturer or the battery manufacturer. The research finds that: (1) For the government, when the vehicle company outsources processing tasks, compared with subsidizing the vehicle company, the total recycling volume when subsidizing the battery manufacturer is higher. When the vehicle company invests in R&D, the total recycling volume under different government subsidy strategies is equal. (2) The vehicle company’s decision is only related to its processing costs; when the unit processing cost is low, the vehicle company’s profit under the strategy of investing in R&D is higher. However, when the unit processing cost is high, the profit of outsourcing processing tasks is higher. (3) With increase in unit subsidy and decrease in unit processing cost, the total recycling volume will increase. These findings can provide decision-making help for the government in formulating subsidy policies and the vehicle company in determining processing strategies in the future.

## 1. Introduction

People’s increasing concern for the environment has recently promoted the rapid development of new energy vehicles (NEVs) around the world [1]. As a key component of NEVs, power batteries have an average service life of 5 to 8 years. Therefore, with the development of the industry, the world will also usher in a peak in the scrapping of power batteries [2,3]. It is estimated that the annual scrap of power batteries in the world will reach 525,000 in 2025 and 1 million in 2030 [4]. Recycling waste batteries has many benefits, including economic, resource and environmental benefits. Firstly, regarding the economic benefits of recycling waste batteries, the research finds that the profit of recycling 1 ton of LMO batteries, 1 ton of LFP batteries, or 1 ton of LCO batteries (LMO, LFP and LCO batteries are three types of lithium-ion batteries) is $431, $196 and $28,016, respectively [5]. Secondly, the significant increase in battery demand leads to a global shortage of key elements [6,7,8], especially cobalt, lithium and nickel, which are non-renewable resources. However, waste batteries contain a large number of metal elements such as cobalt, lithium, nickel and rare earth [9]. Therefore, recycling waste batteries can relieve the supply pressure of key elements and bring resource benefits [10,11,12]. Thirdly, waste batteries contain toxic substances such as toxic electrolytes, organic chemicals and plastics. If they cannot be processed appropriately, they will cause serious harm to the ecological environment and human health [13]. Therefore, recycling waste batteries can bring environmental benefits. Considering the economic benefits of recycling waste batteries, many companies have begun to enter the waste battery recycling and processing market. For example, BMW, Volkswagen and Nissan recycle waste batteries. They use them as home energy storage, and Chevrolet built an energy storage station using waste batteries at the General Motors plant in Michigan [1]. In addition, many companies, such as BYD, BAIC New Energy and GEM, have joined the cascading utilization market [14].

Under the current situation of increasingly fierce competition in the forward supply chain, the reverse supply chain has become a new source of profit for enterprises. However, according to statistics, of the 74,000 tons of power batteries produced in 2018, only 5472 tons were recycled, with a recycling rate of only 7.4%, mainly due to the lack of recycling infrastructure and cost-effective processing technologies. In addition, effective regulations and policies are also of great significance in promoting the recycling of waste batteries and governments around the world are highly concerned about the management of waste battery recycling [15,16]. For example, most states in the United States establish battery recycling channels through industry associations or alliances and require battery manufacturers to adopt designs and labels that facilitate recycling during production. Japan adopts the principle of extended producer responsibility to establish its battery recycling system and South Korea and Germany use the fund and deposit mechanism to establish their recycling systems [17]. In China, the current primary measure is providing subsidies. For example, the Shanghai government stipulates that NEV manufacturers will receive an allowance of 1000 yuan for each waste battery recycled. The Hefei government stipulates that battery manufacturers and NEV manufacturers who recycle waste batteries will be given a subsidy of 10 yuan per kWh based on battery capacity. The Shenzhen government has formulated a deposit refund plan, which stipulates that the government will charge retailers a deposit of 20 yuan per kWh and will refund the retailer a maximum of 10 yuan per kWh as a deposit after recycling waste batteries [15,18].

Although research on recycling and processing waste batteries attracted people’s attention, most studies neglect the decision-making of the companies, i.e., whether companies will enter the waste battery processing market in the future. However, as the competition in the forward supply chain becomes increasingly fierce, more companies will face the issue of whether to outsource processing tasks or invest in R&D. Therefore, the decision-making problem for companies is worth studying. Considering that the battery manufacturers have battery production technology and that the difficulty of using raw materials and recycling and dismantling can be considered in the process of battery R&D, most previous studies regard battery manufacturers as the main body for processing waste batteries. In addition to the battery manufacturer, this paper also regards the vehicle company as the main body of processing waste batteries. The main reasons are as follows: investing in waste battery processing technology requires high cost and vehicle companies are generally in the core position in the reverse supply chain, thus their financial situation is relatively good. Besides, many vehicle enterprises, such as BMW, Volkswagen, BYD, etc., have begun to create a cascade utilization market of waste batteries. Therefore, based on the actual situation, vehicle enterprises can also be the main body for waste batteries processing. At the same time, government subsidies play a guiding role in recycling waste batteries. The selection of government subsidy objects also dramatically impacts the power battery closed-loop supply chain (CLSC). Therefore, in the actual implementation process, how government subsidy can optimize the operation of the CLSC is also worth discussing. Thus, considering the vehicle company outsourcing processing tasks or investing in R&D, this paper studies the optimal decision-making problem of the supply chain under the government or the company subsidizing the battery manufacturer, analyzes the impact of critical parameters on equilibrium decisions and compares equilibrium decisions. This paper intends to solve three problems: (1) From the government level, when the vehicle company determines the method of processing waste batteries, i.e., the vehicle company outsources processing tasks or invests in R&D, which subsidy strategy can the government implement to maximize the total recycling volume of waste batteries? (2) From the level of the vehicle company, when the government determines the subsidy object, i.e., the government subsidizes the battery manufacturer or the vehicle company, which processing decision can the vehicle company take to maximize profits? (3) What is the impact of changes in the unit subsidy and processing costs of the battery manufacturer and the vehicle company on equilibrium decisions?

The contributions of this paper are summarized as follows: (1) Much existing research on waste battery recycling or processing has neglected the problem of waste battery processing decision-making by vehicle companies. This paper considers two situations of vehicle company self-processing and outsourcing processing tasks and studies the decision-making of the vehicle company; (2) Fewer studies have explored the impact of different government subsidy strategies on decision-making in the vehicle company. The findings of this paper can provide decision-making help for the government to formulate subsidy policies and for the vehicle company to determine strategies in the future.

## 2. Literature Review

Research related to this study includes two main aspects: one is research on the decision-making when processing waste batteries by enterprises and the other is research on the impact of government subsidies on recycling waste products.

### 2.1. Research on the Decision-Making in Processing Waste Batteries by Enterprises

Research on decision-making when processing waste batteries by enterprises includes two main aspects: research on enterprise’s investment in in R&D and research on enterprises’ outsourcing of processing tasks. Investing in R&D means that the enterprise is responsible for recycling and processing waste batteries at the same time. Ma et al. [19] designed different contracts to coordinate the waste battery recycling channel; in this model, the automobile manufacturer recycled waste batteries and remanufactured them. Yang et al. [20] studied the impact of different government subsidies on waste battery recycling; in this model, the vehicle company recycled waste batteries and processed them by direct dismantling and cascade utilization.

Outsourcing processing tasks means that the enterprise is only responsible for recycling and outsources processing tasks to other enterprises. Some scholars regard cascade utilization enterprises, recycling and dismantling enterprises, and battery manufacturers as outsourcing enterprises: Zhang and Chen [21] studied the coordination of CLSC under different government subsidy strategies; the third party recycled waste batteries, and batteries were outsourced to battery remanufacturers, cascade utilization and dismantling enterprises, respectively, after identification. Zhao and Ma [22] studied the coordination of waste battery CLSC, in which the third party handed over waste batteries to the battery manufacturer who in turn handed them over to the cascade utilization enterprises and finally to precious metal recycling stations. Some scholars also regard battery manufacturers as outsourcing enterprise: Li and Mu [23] studied the recycling pricing and coordination mechanism of the waste battery CLSC, in which the retailer was responsible for recycling and then outsourced processing tasks to battery manufacturers for remanufacturing or dismantling. Zhu and Li [24] studied the pricing strategy of dual recycling channels for power batteries under different government subsidies; the recycling network and the retailer recycle batteries at the same time and finally outsource them to the automobile manufacturer for remanufacturing. Zhao et al. [25] studied the pricing problem of CLSC for power battery recycling under different recycling strategies, in which the battery manufacturer, retailer or the third party recycled batteries and, finally, the battery manufacturers remanufactured them.

### 2.2. Research on the Impact of Government Subsidies on Recycling Waste Products

Research on the impact of government subsidies on recycling waste products includes two main aspects: research on the impact of different government subsidy policies on recycling waste products and research on the impact of different government subsidy objects on recycling waste products.

Regarding research on the impact of different government subsidy policies on recycling waste products, Zhang et al. [26] studied the impact of government carbon emission reduction subsidies and trade-in subsidies on the CLSC and found that the government should implement carbon emission reduction subsidies to the supply chain, with high carbon emission reduction; on the contrary, the government should implement trade-in subsidies. Wang et al. [27] studied the impact of government initial subsidies, recycling subsidies, R&D subsidies and production subsidies on recycling and remanufacturing activities and the study found that different subsidy methods are suitable for different development stages of the remanufacturing supply chain. Besides, compared with a single subsidy policy, mixed subsidy policies have a better promotion effect on remanufacturing. Wang and Deng [28] compared the decision-making of the reverse supply chain under anarchic intervention, government rewards and punishments and tax-subsidy and the study found that the reward and punishment mechanism is more effective than the tax-subsidy mechanism in guiding members to recycle and remanufacture waste products. Li et al. [29] compared the impact of government subsidy policy and carbon tax policy on authorized remanufacturing.

Regarding research on the impact of different government subsidy objects on recycling waste products, Ma et al. [30] studied the impact of government subsidies to the consumer on members of the CLSC with dual recycling channels. Yao et al. [31] and Zhou et al. [32] studied the CLSC decision-making problem under the government’s remanufacturing subsidy to manufacturers. Zhao and Lin [33] studied the CLSC pricing model under different subsidy objects and the study found that, when the government subsidizes the consumer or the retailer, the pricing index on the sales channel is higher and, when the government subsidizes the manufacturer and the third party, the pricing index on the recycling channel and the profit of members are higher. Zhao et al. [34] studied the impact of government subsidies to the recycler and the processor on waste products recycling under classified recycling. Liu and Ma [35] studied the influence of non-subsidy, subsidized recyclers, and cascade utilization by the enterprise and the manufacturer on CLSC and found that the government should subsidize the recycler or the manufacturer in the early stage and the cascade utilization enterprise in the later stage.

## 3. The Model

### 3.1. Problem Description

**Figure 1:**
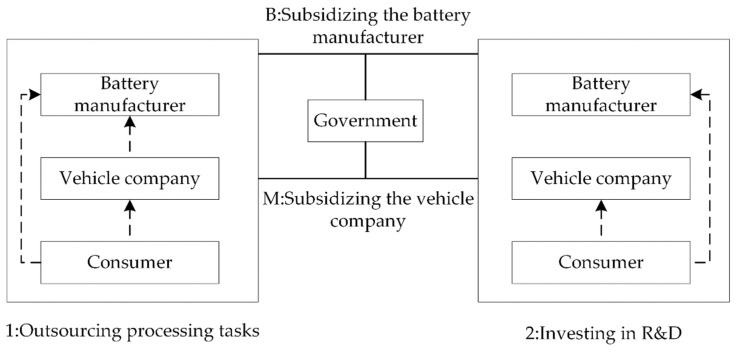
The influence of different government subsidy strategies on the vehicle company’s decision-making in processing waste batteries.

The main research object of this paper is the supply chain composed of a battery manufacturer and a vehicle company, as shown in Figure 1. Both the battery manufacturer and the vehicle company can recycle waste batteries. Besides, the battery manufacturer is not only responsible for recycling but can also benefit from processing waste batteries and the vehicle company has two strategies: outsourcing processing tasks or investing in R&D. In order to promote enterprises in recycling waste batteries, the government has two subsidy strategies: subsidizing the battery manufacturer or subsidizing the vehicle company. The decision-making process of the battery manufacturer and the vehicle company is as follows: when the vehicle company outsources processing tasks, first the battery manufacturer decides the commissioned recycling price of waste batteries, then the battery manufacturer and the vehicle company respectively decide their own recycling price for waste batteries according to the commissioned recycling price; When the vehicle company invests in R&D, the battery manufacturer and the vehicle company only need to decide their own recycling price for waste batteries.

### 3.2. Model Assumptions

First, the following assumptions are made for the model:

(1) In most cases, vehicle companies are “assembly plants” that do not have technologies for battery production, disassembly and processing, while battery manufacturers have battery production technology and can consider the use of raw materials and the difficulty of recycling and dismantling in the process of battery R&D. Therefore, battery manufacturers have more advantages in processing waste batteries [36]. Considering the above situations, this paper assumes that the battery manufacturer has the ability to process waste batteries in the outsourcing processing mode, assuming that the vehicle manufacturer outsources processing tasks to the battery manufacturer, and in the investing in R&D mode, assuming that the vehicle company invests in R&D and processes waste batteries by itself.

(2) Referring to the function of the recycling volume of waste products by some scholars under mixed channels, it is assumed that the recycling volume of waste batteries by the battery manufacturer and the vehicle company is respectively [37]:

$$D_{B}^{ij}=r_{0}+r_{1}p_{B}^{ij}-r_{2}p_{M}^{ij},\;D_{M}^{ij}=r_{0}+r_{1}p_{M}^{ij}-r_{2}p_{B}^{ij},\;$$

where i={B,M}, j={1,2}.

The parameters involved in this paper are shown in Nomenclature.

### 3.3. Model Analysis

#### 3.3.1. Outsourcing Processing Tasks When Subsidizing the Battery Manufacturer

When the government subsidizes the battery manufacturer and the vehicle company outsources processing tasks to the battery manufacturer, based on the above assumptions the profits of the battery manufacturer and the vehicle company are expressed as:

$$\pi_{B}^{B1}=(f-p_{B}^{B1}-c_{1}+s)(r_{0}+r_{1}p_{B}^{B1}-r_{2}p_{M}^{B1})+(f-w^{B}-c_{1}+s)(r_{0}+r_{1}p_{M}^{B1}-r_{2}p_{B}^{B1})\;\pi_{M}^{B1}=(w^{B}-p_{M}^{B1})(r_{0}+r_{1}p_{M}^{B1}-r_{2}p_{B}^{B1})$$

The equilibrium decisions are solved as:

$$w^{B}{}^{*}=\frac{-r_{0}(8r_{1}^{3}+r_{2}^{3})+(r_{1}-r_{2})(8r_{1}^{3}+2r_{1}r_{2}^{2}-r_{2}^{3})(f-c_{1}+s)}{2r_{1}(r_{1}-r_{2})(8r_{1}^{2}+r_{2}^{2})},\;p_{B}^{B1}{}^{*}=\frac{-r_{0}(2r_{1}+r_{2})+2r_{1}(r_{1}-r_{2})(f-c_{1}+s)+3r_{1}r_{2}w^{B}{}^{*}}{4r_{1}^{2}-r_{2}^{2}};\;p_{M}^{B1}{}^{*}=\frac{-r_{0}(2r_{1}+r_{2})+r_{2}(r_{1}-r_{2})(f-c_{1}+s)+(2r_{1}^{2}+r_{2}^{2})w^{B}{}^{*}}{4r_{1}^{2}-r_{2}^{2}},\;D_{B}^{B1}{}^{*}=\frac{r_{0}r_{1}(2r_{1}+r_{2})+(r_{1}-r_{2})(2r_{1}^{2}-r_{2}^{2})(f-c_{1}+s)+r_{2}(r_{1}^{2}-r_{2}^{2})w^{B}{}^{*}}{4r_{1}^{2}-r_{2}^{2}};\;D_{M}^{B1}{}^{*}=\frac{r_{1}[r_{0}(2r_{1}+r_{2})-r_{2}(r_{1}-r_{2})(f-c_{1}+s)+2(r_{1}^{2}-r_{2}^{2})w^{B}{}^{*}]}{4r_{1}^{2}-r_{2}^{2}},\;D^{B1}{}^{*}=\frac{2r_{0}r_{1}+{\left(r_{1}-r_{2}\right)}^{2}(f-c_{1}+s)+(r_{1}^{2}-r_{2}^{2})w^{B}{}^{*}}{2r_{1}-r_{2}};\;\pi_{B}^{B1}{}^{*}=\frac{[r_{0}(2r_{1}+r_{2})+(2r_{1}^{2}+2r_{1}r_{2}-r_{2}^{2})(f-c_{1}+s)-3r_{1}r_{2}w^{B}{}^{*}]D_{B}^{B1}{}^{*}}{4r_{1}^{2}-r_{2}^{2}}+(f-w^{B}{}^{*}-c_{1}+s)D_{M}^{B1}{}^{*};\;\pi_{M}^{B1}{}^{*}=\frac{r_{1}{\left[r_{0}(2r_{1}+r_{2})-r_{2}(r_{1}-r_{2})(f-c_{1}+s)+2(r_{1}^{2}-r_{2}^{2})w^{B}{}^{*}\right]}^{2}}{{\left(4r_{1}^{2}-r_{2}^{2}\right)}^{2}}.\;$$

#### 3.3.2. Investing in R&D When Subsidizing the Battery Manufacturer

When the government subsidizes the battery manufacturer and the vehicle company invests in R&D, based on the above assumptions the profits of the battery manufacturer and the vehicle company are expressed as:

$$\pi_{B}^{B2}=(f-p_{B}^{B2}-c_{1}+s)(r_{0}+r_{1}p_{B}^{B2}-r_{2}p_{M}^{B2})\;\pi_{M}^{B2}=(f-p_{M}^{B2}-c_{2})(r_{0}+r_{1}p_{M}^{B2}-r_{2}p_{B}^{B2})$$

The equilibrium decisions are solved as:

$$p_{B}^{B2}{}^{*}=\frac{-r_{0}(2r_{1}+r_{2})-r_{1}(2c_{1}r_{1}+c_{2}r_{2})+r_{1}(2r_{1}+r_{2})f+2r_{1}^{2}s}{4r_{1}^{2}-r_{2}^{2}},\;p_{M}^{B2}{}^{*}=\frac{-r_{0}(2r_{1}+r_{2})-r_{1}(c_{1}r_{2}+2c_{2}r_{1})+r_{1}(2r_{1}+r_{2})f+r_{1}r_{2}s}{4r_{1}^{2}-r_{2}^{2}};\;D_{B}^{B2}{}^{*}=\frac{r_{1}[r_{0}(2r_{1}+r_{2})-2c_{1}r_{1}^{2}+c_{2}r_{1}r_{2}+c_{1}r_{2}^{2}+(r_{1}-r_{2})(2r_{1}+r_{2})f+(2r_{1}^{2}-r_{2}^{2})s]}{4r_{1}^{2}-r_{2}^{2}};\;D_{M}^{B2}{}^{*}=\frac{r_{1}[r_{0}(2r_{1}+r_{2})-2c_{2}r_{1}^{2}+c_{1}r_{1}r_{2}+c_{2}r_{2}^{2}+(r_{1}-r_{2})(2r_{1}+r_{2})f-r_{1}r_{2}s]}{4r_{1}^{2}-r_{2}^{2}},\;D^{B2}{}^{*}=\frac{r_{1}[2r_{0}+(r_{1}-r_{2})(2f-c_{1}-c_{2}+s)]}{2r_{1}-r_{2}};\;\pi_{B}^{B2}{}^{*}=\frac{r_{1}{\left[r_{0}(2r_{1}+r_{2})-2c_{1}r_{1}^{2}+c_{2}r_{1}r_{2}+c_{1}r_{2}^{2}+(r_{1}-r_{2})(2r_{1}+r_{2})f+(2r_{1}^{2}-r_{2}^{2})s\right]}^{2}}{{\left(4r_{1}^{2}-r_{2}^{2}\right)}^{2}};\;\pi_{M}^{B2}{}^{*}=\frac{r_{1}{\left[r_{0}(2r_{1}+r_{2})-2c_{2}r_{1}^{2}+c_{1}r_{1}r_{2}+c_{2}r_{2}^{2}+(r_{1}-r_{2})(2r_{1}+r_{2})f-r_{1}r_{2}s\right]}^{2}}{{\left(4r_{1}^{2}-r_{2}^{2}\right)}^{2}}.\;$$

#### 3.3.3. Outsourcing Processing Tasks When Subsidizing the Vehicle Company

When the government subsidizes the vehicle company and the vehicle company outsources processing tasks to the battery manufacturer, based on the above assumption, the profits of the battery manufacturer and the vehicle company are expressed as:

$$\pi_{B}^{M1}=(f-p_{B}^{M1}-c_{1})(r_{0}+r_{1}p_{B}^{M1}-r_{2}p_{M}^{M1})+(f-w^{M}-c_{1})(r_{0}+r_{1}p_{M}^{M1}-r_{2}p_{B}^{M1})\;\pi_{M}^{M1}=(w-p_{M}^{M1}+s)(r_{0}+r_{1}p_{M}^{M1}-r_{2}p_{B}^{M1})$$

The equilibrium decisions are solved as:

$$w^{M}{}^{*}=\frac{-r_{0}(8r_{1}^{3}+r_{2}^{3})+(r_{1}-r_{2})(8r_{1}^{3}+2r_{1}r_{2}^{2}-r_{2}^{3})(f-c_{1})-8r_{1}^{3}s(r_{1}-r_{2})}{2r_{1}(r_{1}-r_{2})(8r_{1}^{2}+r_{2}^{2})},\;p_{B}^{M1}{}^{*}=\frac{-r_{0}(2r_{1}+r_{2})+2r_{1}(r_{1}-r_{2})(f-c_{1})+r_{1}r_{2}s+3r_{1}r_{2}w^{M}{}^{*}}{4r_{1}^{2}-r_{2}^{2}};\;p_{M}^{M1}{}^{*}=\frac{-r_{0}(2r_{1}+r_{2})+r_{2}(r_{1}-r_{2})(f-c_{1})+2r_{1}^{2}s+(2r_{1}^{2}+r_{2}^{2})w^{M}{}^{*}}{4r_{1}^{2}-r_{2}^{2}};\;D_{B}^{M1}{}^{*}=\frac{r_{0}r_{1}(2r_{1}+r_{2})+(r_{1}-r_{2})(2r_{1}^{2}-r_{2}^{2})(f-c_{1})-r_{1}^{2}r_{2}s+r_{2}(r_{1}^{2}-r_{2}^{2})w^{M}{}^{*}}{4r_{1}^{2}-r_{2}^{2}};\;D_{M}^{M1}{}^{*}=\frac{r_{1}[r_{0}(2r_{1}+r_{2})-r_{2}(r_{1}-r_{2})(f-c_{1})+(2r_{1}^{2}-r_{2}^{2})s+2(r_{1}^{2}-r_{2}^{2})w^{M}{}^{*}]}{4r_{1}^{2}-r_{2}^{2}};\;D^{M1}{}^{*}=\frac{2r_{0}r_{1}+{\left(r_{1}-r_{2}\right)}^{2}(f-c_{1})+r_{1}(r_{1}-r_{2})s+(r_{1}^{2}-r_{2}^{2})w^{B}{}^{*}}{2r_{1}-r_{2}};\;\pi_{B}^{M1}{}^{*}=\frac{[r_{0}(2r_{1}+r_{2})+(2r_{1}^{2}+2r_{1}r_{2}-r_{2}^{2})(f-c_{1})-r_{1}r_{2}s-3r_{1}r_{2}w^{M}{}^{*}]D_{B}^{M1}{}^{*}}{{\left(4r_{1}^{2}-r_{2}^{2}\right)}^{2}}+(f-w^{M}{}^{*}-c1)D_{M}^{M1}{}^{*};\;\pi_{M}^{M1}{}^{*}=\frac{r_{1}{\left[r_{0}(2r_{1}+r_{2})-r_{2}(r_{1}-r_{2})(f-c_{1})+(2r_{1}^{2}-r_{2}^{2})s+2(r_{1}^{2}-r_{2}^{2})w^{M}{}^{*}\right]}^{2}}{{\left(4r_{1}^{2}-r_{2}^{2}\right)}^{2}}.\;$$

#### 3.3.4. Investing in R&D When Subsidizing the Vehicle Company

When the government subsidizes the vehicle company and the vehicle company invests in R&D, based on the above assumptions the profits of the battery manufacturer and the vehicle company are expressed as:

$$\pi_{B}^{M2}=(f-p_{B}^{M2}-c_{1})(r_{0}+r_{1}p_{B}^{M2}-r_{2}p_{M}^{M2})\;\pi_{M}^{M2}=(f-p_{M}^{M2}-c_{2}+s)(r_{0}+r_{1}p_{M}^{M2}-r_{2}p_{B}^{M2})$$

The equilibrium decisions are solved as:

$$p_{B}^{M2}{}^{*}=\frac{-r_{0}(2r_{1}+r_{2})-r_{1}(2c_{1}r_{1}+c_{2}r_{2})+r_{1}(2r_{1}+r_{2})f+r_{1}r_{2}s}{4r_{1}^{2}-r_{2}^{2}},\;p_{M}^{M2}{}^{*}=\frac{-r_{0}(2r_{1}+r_{2})-r_{1}(c_{1}r_{2}+2c_{2}r_{1})+r_{1}(2r_{1}+r_{2})f+2r_{1}^{2}s}{4r_{1}^{2}-r_{2}^{2}};\;D_{B}^{M2}{}^{*}=\frac{r_{1}[r_{0}(2r_{1}+r_{2})-2c_{1}r_{1}^{2}+c_{2}r_{1}r_{2}+c_{1}r_{2}^{2}+(r_{1}-r_{2})(2r_{1}+r_{2})f-r_{1}r_{2}s]}{4r_{1}^{2}-r_{2}^{2}};\;D_{M}^{M2}{}^{*}=\frac{r_{1}[r_{0}(2r_{1}+r_{2})-2c_{2}r_{1}^{2}+c_{1}r_{1}r_{2}+c_{2}r_{2}^{2}+(r_{1}-r_{2})(2r_{1}+r_{2})f+(2r_{1}^{2}-r_{2}^{2})s]}{4r_{1}^{2}-r_{2}^{2}};\;D^{M2}{}^{*}=\frac{r_{1}[2r_{0}+(r_{1}-r_{2})(2f-c_{1}-c_{2}+s)]}{2r_{1}-r_{2}},\;\pi_{B}^{M2}{}^{*}=\frac{r_{1}{\left[r_{0}(2r_{1}+r_{2})-2c_{1}r_{1}^{2}+c_{2}r_{1}r_{2}+c_{1}r_{2}^{2}+(r_{1}-r_{2})(2r_{1}+r_{2})f-r_{1}r_{2}s\right]}^{2}}{{\left(4r_{1}^{2}-r_{2}^{2}\right)}^{2}};\;\pi_{M}^{M2}{}^{*}=\frac{r_{1}{\left[r_{0}(2r_{1}+r_{2})-2c_{2}r_{1}^{2}+c_{1}r_{1}r_{2}+c_{2}r_{2}^{2}+(r_{1}-r_{2})(2r_{1}+r_{2})f+(2r_{1}^{2}-r_{2}^{2})s\right]}^{2}}{{\left(4r_{1}^{2}-r_{2}^{2}\right)}^{2}}.$$

## 4. Comparative Analysis

In the previous section, we built and solved four models that considered government subsidy strategies and the vehicle company’s processing decisions. Next, we discuss the research results from two aspects: sensitivity analysis and equilibrium decision analysis.

### 4.1. Sensitivity Analysis

In this subsection, we analyze the impact of the unit subsidy s, the battery manufacturer’s unit processing cost $c_{1}$ and the vehicle company’s unit processing cost $c_{2}$ on equilibrium decisions under four models.

Table 1 shows the impact of the unit subsidy on equilibrium decisions under four models. It can be seen from Table 1 that: (1) With the increase in the unit subsidy, the total recycling volume of waste batteries under four models will increase. This shows that, no matter what strategy the government and the vehicle company take, government subsidies can always increase the total recycling volume of waste batteries. This is consistent with some existing studies [20,21,35], i.e., government subsidies can increase the total recycling volume of waste batteries. The difference is that this paper further studies the influence of government subsidies on the total recycling volume of waste batteries under different government subsidy objects and different processing decisions of vehicle companies and considers the situation more comprehensively. (2) With the increase in the unit subsidy, the equilibrium decisions under Model B1 will increase, while the commissioned recycling price, the recycling price and the recycling volume of waste batteries will decrease under Model M1. This shows that it is beneficial for the government to subsidize the battery manufacturer when the vehicle company outsources processing tasks. However, when the government subsidizes the vehicle company, with the increase of the unit subsidy, the battery manufacturer will divide the subsidies received by the vehicle manufacturer by reducing the commissioned recycling price and the enthusiasm of the battery manufacturer will also be reduced. This is consistent with the research [24]. In Model B1 and Model M1, the vehicle company outsources recycled waste batteries to the battery manufacturer. At this time, the battery manufacturer is the leader of the Stackelberg game and the vehicle company is a follower, which shows that, when the government subsidizes the battery manufacturer, the battery manufacturer will increase the commissioned recycling price of waste batteries. When the government subsidizes the vehicle company, the battery manufacturer will reduce the commissioned recycling price. (3) With the increase in the unit subsidy, the recycling volume and the profit of the vehicle manufacturer under Model B2 will decrease and the recycling volume and the profit of the battery manufacturer under Model M2 will decrease. This is because, when the vehicle company invests in R&D, the battery manufacturer and the vehicle company form perfect competition and subsidies to the battery manufacturer are unfavorable for the vehicle company. Similarly, subsidies to the vehicle company are unfavorable for the battery manufacturer. This is consistent with research [32], i.e., the competition among supply chain members will reduce the recovery rate of waste products, which is not conducive to the development of the supply chain.

Table 2 shows the impact of the battery manufacturer’s unit processing cost $c_{1}$ and the vehicle manufacturer’s unit processing cost $c_{2}$ on equilibrium decisions under four models. It can be seen from Table 2 that: (1) With the increase in the unit processing cost, the total recycling volume of waste batteries under four models will decrease. (2) With the increase in the battery manufacturer’s unit processing cost, equilibrium decisions in Model B1 and Model M1 will decrease. This is because, in Model B1 and Model M1, the vehicle company and the battery manufacturer are a community of interests and the increase in the unit processing cost of the battery manufacturer will have a negative effect on the vehicle company. (3) With the increase in the battery manufacturer’s unit processing cost, the recycling volume and the profit of the vehicle company in Model B2 and Model M2 will increase. Similarly, with the increase in the vehicle company’s unit processing cost, the recycling volume and the profit of the battery manufacturer in Model B2 and Model M2 will increase. This is because in Model B2 and Model M2, the battery manufacturer and the vehicle company form perfect competition and the increase in the battery manufacturer’s unit processing cost is beneficial to the vehicle company. Similarly, the increase in the vehicle company’s unit processing cost is also beneficial for the battery manufacturer. This is consistent with research [23], i.e., with the increase of the processing cost of unit waste battery, the total recycling volume of waste batteries will also decrease and the impact of the increase of processing cost on the profits of each member of the supply chain is different.

### 4.2. Equilibrium Decisions Analysis

In this subsection, we conduct a comparative analysis of equilibrium decisions under four models. Firstly, under the fixed processing strategy, the total recycling volume of waste batteries and the profit of the vehicle company under different government subsidy objects are compared, as shown in Table 3. Secondly, under the fixed government object, the total recycling volume of waste batteries and the profit of the vehicle company under the vehicle company’s different processing strategies are compared, as shown in Table 4.

It can be seen from Table 3 that, regardless of whether the vehicle company outsources processing tasks or invests in R&D, the profit of the vehicle company is higher when the government subsidizes the vehicle company. However, when the vehicle company outsources processing tasks, the total recycling volume of waste batteries is higher when the government subsidizes the battery manufacturer. This is consistent with the research [33], i.e., different government subsidy objects have different impacts on recycling channels. However, when the vehicle company invests in R&D, the total recycling volume of waste batteries under different government subsidy objects is equal. This is consistent with the research [34], i.e., different government subsidy objects only have an impact on the indirect recycling price of waste products, but have the same effect on the total recycling amount of waste products, because when the vehicle company outsources the processing of waste batteries to the battery manufacturer, the battery manufacturer is the leader of the supply chain. At this time, government subsidies to the battery manufacturer can significantly increase the total recycling volume of waste batteries. However, when the vehicle company invests in R&D to process waste batteries, the battery manufacturer and the vehicle company have the same position in the reverse supply chain. At this time, government subsidies will only change the recycling volume of each member, but have no effect on the total recycling volume of waste batteries.

It can be seen from Table 4 that no matter whether the government subsidizes the vehicle company or the battery manufacturer, when the unit processing cost of the vehicle company is low, the total recycling volume of waste batteries and the profit of the vehicle company under the strategy of investing in R&D are higher. Conversely, when the unit processing cost of the vehicle company is relatively high, the total recycling volume of waste batteries and the profit of the vehicle company under the strategy of outsourcing processing tasks are higher.

## 5. Example Analysis

In this section, an example will be used to simulate the impact of different government subsidy strategies on the total recycling volume of waste batteries $D^{ij*}$ and the profit of the vehicle company $\pi_{M}^{ij*}$ under two situations the vehicle company outsources processing tasks or invests in R&D. The corresponding parameter values are set as: f=100, $c_{1}=30$, $c_{2}=40$, $r_{0}=20$, $r_{1}=4$, $r_{2}=1$, s=0~10.

### 5.1. The Impact of Different Subsidy Strategies on $D^{ij*}$ and $\pi_{M}^{ij*}$ under the Fixed Processing Strategy

**Figure 2:**
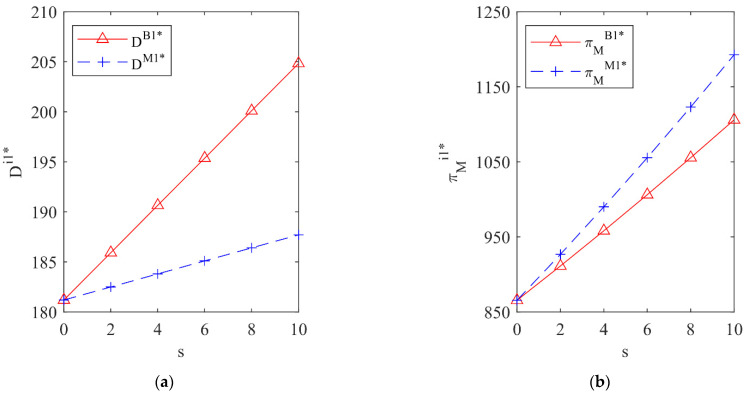
The influence of different subsidy strategies on $D^{i1*}$ and $\pi_{M}^{i1*}$ under outsourcing processing tasks. (**a**) The total recycling volume; (**b**) The profit of the vehicle company.

Figure 2 shows the total recycling volume of waste batteries and the profit of the vehicle company under the government subsidizing the battery manufacturer or the vehicle company when the vehicle company outsources processing tasks. It can be seen from Figure 2 that, when the vehicle company outsources processing tasks compared with subsidizing the vehicle company, the total recycling volume of waste batteries under the government subsidizing the battery manufacturer is higher, but the profit of the vehicle company is lower. Therefore, from the perspective of maximizing the total recycling volume of waste batteries, the government should subsidize the battery manufacturer.

**Figure 3:**
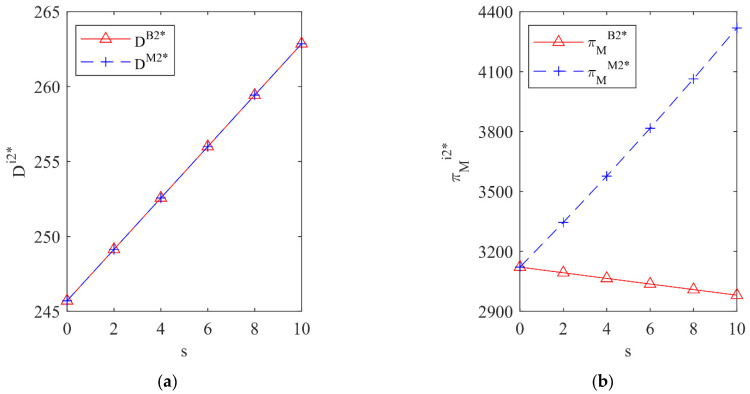
The influence of different subsidy strategies on $D^{i2*}$ and $\pi_{M}^{i2*}$ under investing in R&D. (**a**) The total recycling volume; (**b**) The profit of the vehicle company.

Figure 3 shows the total recycling volume of waste batteries and the profit of the vehicle company under the government subsidizing the battery manufacturer or the vehicle company when the vehicle company invests in R&D. It can be seen from Figure 3 that, when the vehicle company invests in R&D, the total recycling volume of waste batteries under different government subsidy objects is the same and the profit of the vehicle company is higher when the government subsidizes the vehicle company. Therefore, from the perspective of pursuing the maximization of the total recycling volume of waste batteries and the higher enthusiasm of the vehicle company for recycling, the government should subsidize the vehicle company.

### 5.2. The Impact of Different Subsidy Strategies on $D^{ij*}$ and $\pi_{M}^{ij*}$ under the Fixed Subsidy Strategy

**Figure 4:**
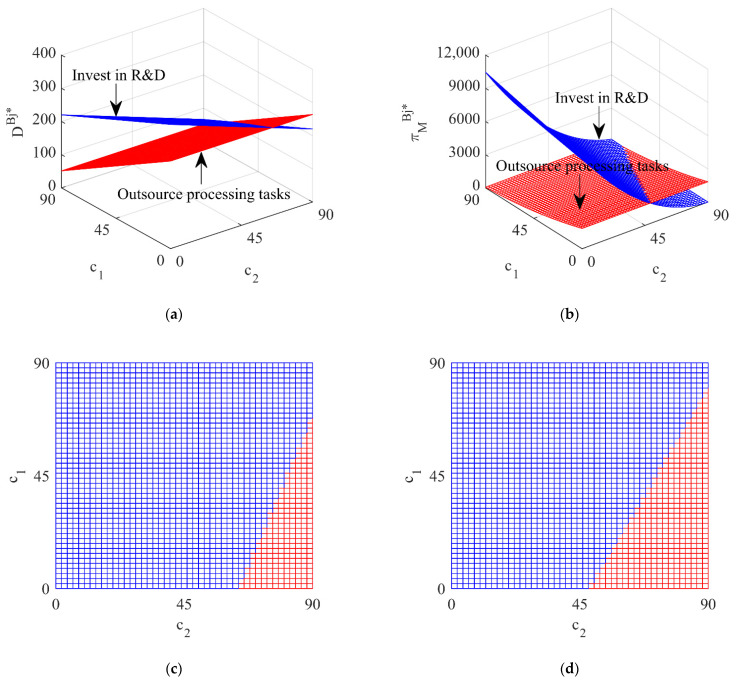
The influence of different processing strategies on $D^{\mathrm{Bj}*}$ and $\pi_{M}^{\mathrm{Bj}*}$ under subsidizing the battery manufacturer. (**a**) The total recycling volume; (**b**) The profit of the vehicle company; (**c**) Top view of the total recycling volume; (**d**) Top view of the profit of the vehicle company.

**Figure 5:**
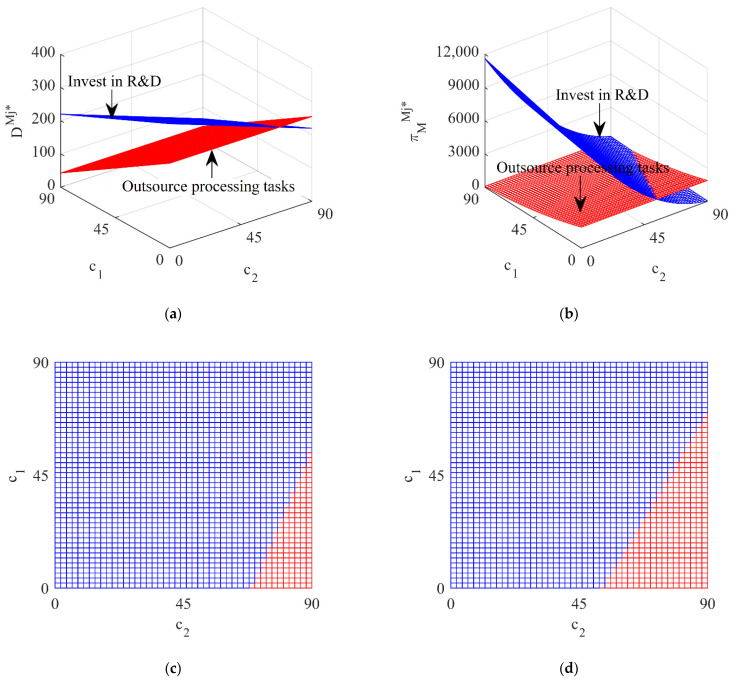
The influence of different processing strategies on $D^{\mathrm{Mj}*}$ and $\pi_{M}^{\mathrm{Mj}*}$ under subsidizing the vehicle company. (**a**) The total recycling volume; (**b**) The profit of the vehicle company; (**c**) Top view of the total recycling volume; (**d**) Top view of the profit of the vehicle company.

Figure 4 shows the impact of different processing strategies of the vehicle company under the government subsidizing the battery manufacturer and Figure 5 shows the impact of different processing strategies of the vehicle company under the government subsidizing the vehicle company.

From Figure 4 and Figure 5, it can be seen that, whether the government subsidizes the battery manufacturer or the vehicle company, when the unit processing cost of the vehicle company is low (blue area), the total recycling volume of waste batteries and the profit of the vehicle company under the vehicle company investing in R&D is higher than for outsourcing processing tasks, so for the sake of profit maximization the vehicle company will invest in R&D; when the unit processing cost of the vehicle company is high (red area), the total recycling volume of waste batteries and the profit of the vehicle company under the vehicle company outsourcing processing tasks is higher and the vehicle company will outsource processing tasks. This shows that different government subsidy strategies have little impact on the decision-making of the vehicle company and the processing strategy of the vehicle company is mainly affected by its own unit processing cost.

## 6. Conclusions and Policy Recommendations

### 6.1. Conclusions

Firstly, the research on the decision-making of processing waste batteries by enterprises includes two main aspects: research on enterprise’s investing in R&D [19,20] and on enterprises’ outsourcing processing tasks [21,22,23,24,25], but these studies only consider the single processing mode. Secondly, many researchers have studied the waste battery recycling supply chain under government subsidies, including different government subsidy policies [26,27,28,29] and objects [30,31,32,33,34,35]. However, these researchers only consider the impact of subsidies on the recycling amount of waste batteries and the profits of supply chain members, while ignoring the impact of government subsidies on enterprises’ decision-making when processing waste batteries. In fact, the vehicle company’s company is diverse and the government’s subsidy strategy and the vehicle company’s decision-making to process waste batteries influence each other. Therefore, considering the vehicle company outsourcing processing tasks or investing in R&D, this paper studies the optimal decisions of the supply chain under the government subsidizing the battery manufacturer or the vehicle company. The findings of this paper can provide decision-making help for the government to formulate subsidy policies and for the vehicle company to determine strategies in the future. Through theoretical analysis and example analysis, the results show that: (1) For the government, when the vehicle company outsources processing tasks compared with subsidizing the vehicle company, the total recycling volume of waste batteries under subsidizing the battery manufacturer is higher, but the profit of the vehicle company is lower; When the vehicle company invests in R&D, the total recycling volume under different government subsidy strategies is equal, but the profit is higher under subsidizing the vehicle company. (2) The vehicle company’s decision is only related to its processing costs, when the unit processing cost is low; under the strategy of investing in R&D, the total recycling volume of waste batteries and the profit of the vehicle company are higher; When the unit processing cost is high, under the strategy of outsourcing processing tasks, the total recycling volume and the profit are higher. (3) With the increase in the unit subsidy and the decrease in the unit processing cost, the total recycling volume of waste batteries under four models will increase.

### 6.2. Policy Recommendations

In addition, this paper also puts forward relevant policy recommendations for the government and enterprises based on the above research results. The practical implications for the government are as follows: (1) When there is only a single company processing waste batteries in the supply chain, the government should subsidize this company; when there are multiple companies processing waste batteries, the government can subsidize any company responsible for processing waste batteries; (2) High processing costs are the main factor that hinders enterprises from processing waste batteries. Therefore, the government can consider subsidizing enterprises based on their processing costs so as to encourage enterprises to process waste batteries.

The practical implications for enterprises are as follows: (1) Due to the high cost of processing waste batteries, vehicle companies with insufficient funds should not rashly enter the processing market. They can continue to deploy the recycling market and then outsource processing tasks to professional processing agencies; (2) For vehicle companies with relatively abundant funds, it is possible to consider establishing a waste battery processing branch by themselves. At the same time, it is also necessary to pay attention to improving the technology of processing waste batteries and reducing the unit processing cost; (3) For battery manufacturers, they can consider the recycling and processing of waste batteries at the beginning of power battery design, such as designing power batteries with higher safety, easier disassembly and lower content of toxic substances, which will be convenient for battery manufacturers in recycling waste batteries and will also reduce processing costs.

### 6.3. Limitations and Future Research

This paper has some limitations. The model constructed is composed of a single battery manufacturer and a single vehicle company. In reality, third-party recyclers, secondary utilization companies and recycling companies are in the power battery supply chain. Therefore, further research should consider a supply chain that contains more subjects. In addition, to examine the battery recycling and processing market in real life, many companies have begun to explore ways to recycle and process waste batteries cooperatively to obtain higher profits. Therefore, the cooperation of supply chain entities can be further considered in further research. Finally, in addition to the subsidy policy, the government has also formulated other approaches to increase the recycling volume of waste batteries, such as deposit refunds, government rewards and punishment, etc. Therefore, in further research, the impact of different government policies on recycling waste batteries can also be considered.

## Figures and Tables

**Table 1:** The influence of the unit subsidy s on equilibrium decisions under four models.

Model	Decision							
	w	$p_{B}$	$p_{M}$	$D_{B}$	$D_{M}$	D	$\pi_{B}$	$\pi_{M}$
B1	↑	↑	↑	↑	↑	↑	↑	↑
M1	↓	↓	↑	↓	↑	↑	↑	↑
B2	N/A	↑	↑	↑	↓	↑	↑	↓
M2	N/A	↑	↑	↓	↑	↑	↓	↑

Note: ↑: increasing; ↓: decreasing; N/A: Not Applicable.

**Table 2:** The influence of unit processing cost $c_{1}$, $c_{2}$ on equilibrium decisions under four models.

Model	Parameters	Decision							
		w	$p_{B}$	$p_{M}$	$D_{B}$	$D_{M}$	D	$\pi_{B}$	$\pi_{M}$
B1	$c_{1}$	↓	↓	↓	↓	↓	↓	↓	↓
M1	$c_{1}$	↓	↓	↓	↓	↓	↓	↓	↓
B2	$c_{1}$	↓	↓	↓	↓	↑	↓	↓	↑
	$c_{2}$	↓	↓	↓	↑	↓	↓	↑	↓
M2	$c_{1}$	↓	↓	↓	↓	↑	↓	↓	↑
	$c_{2}$	↓	↓	↓	↑	↓	↓	↑	↓

Note: ↑: increasing; ↓: decreasing.

**Table 3:** Comparison of government subsidy strategies under the fixed processing strategy.

	Outsource Processing Tasks	Invest in R&D
$D^{ij*}$	$D^{B1}{}^{*}>D^{M1}{}^{*}$	$D^{B2}{}^{*}=D^{M2}{}^{*}$
$\pi_{M}^{ij*}$	$\pi_{M}^{B1}{}^{*}<\pi_{M}^{M1}{}^{*}$	$\pi_{M}^{B2}{}^{*}<\pi_{M}^{M2}{}^{*}$

**Table 4:** Comparison of the vehicle company’s processing strategies under the fixed subsidy strategy.

Subsidize the Battery Manufacturer		Subsidize the Vehicle Company	
Condition	Decision	Condition	Decision
$0<c_{2}<\min\{a_{1},a_{2},a_{3},a_{4}\}$	$D^{B1*}<D^{B2}{}^{*}$	$0<c_{2}<\min\{b_{1},b_{2},b_{3},b_{4}\}$	$D^{M1*}<D^{M2}{}^{*}$
$a_{4}<c_{2}<\min\{a_{1},a_{2},a_{3}\}$	$D^{B1*}>D^{B2}{}^{*}$	$b_{4}<c_{2}<\min\{b_{1},b_{2},b_{3}\}$	$D^{M1*}>D^{M2}{}^{*}$
$0<c_{2}<\min\{a_{1},a_{2},a_{5}\}$	$\pi_{M}^{B1*}<\pi_{M}^{B2*}$	$0<c_{2}<\min\{b_{1},b_{2},b_{5}\}$	$\pi_{M}^{M1*}<\pi_{M}^{M2*}$
$a_{5}<c_{2}<\min\{a_{1},a_{2},a_{3}\}$	$\pi_{M}^{B1*}>\pi_{M}^{B2*}$	$b_{5}<c_{2}<\min\{b_{1},b_{2},b_{3}\}$	$\pi_{M}^{M1*}>\pi_{M}^{M2*}$

## Data Availability

Not applicable.

## Nomenclature

i	government subsidy strategies, i={B,M}, where i=B means subsidizing the battery manufacturer, i=M means subsidizing the vehicle company
j	ways to process waste batteries by the vehicle company, j={1,2}, where j=1 means outsourcing processing tasks, j=2 means investing in R&D
s	unit subsidy of the government
f	profit from processing unit waste battery
$c_{1}$	processing cost of unit waste battery by the battery manufacturer
$c_{2}$	processing cost of unit waste battery by the vehicle company
$w^{i}$	the commissioned recycling price of waste batteries by the battery manufacturer
$p_{B}^{ij}$	the recycling price of waste batteries by the battery manufacturer
$p_{M}^{ij}$	the recycling price of waste batteries by the vehicle company
$D_{B}^{ij}$	the recycling volume of waste batteries by the battery manufacturer
$D_{M}^{ij}$	the recycling volume of waste batteries by the vehicle company
$D^{ij}$	the total recycling volume of waste batteries
$\pi_{B}^{ij}$	the profit of the battery manufacturer
$\pi_{M}^{ij}$	the profit of the vehicle company
$r_{0}$	the number of recycled power batteries based on consumers’ environmental awareness
$r_{1}$	consumer sensitivity to recycling prices of waste batteries
$r_{2}$	sensitivity of consumers to recycling prices of waste batteries in different recycling channels, $r_{1}>r_{2}>0$
